# Genetic diversity in F3 segregating populations of rice (*Oryza sativa* L.) genotypes under salt stress

**DOI:** 10.3389/fpls.2025.1568859

**Published:** 2025-04-08

**Authors:** Azhar Ali Laghari, Aqeel Ahmad, Shabana Memon, Syed Abdul Majeed Musavi, Aamir Ali, Akash Kumar, Qingxia Guo

**Affiliations:** ^1^ College of Resources and Environment, Shanxi Agricultural University, Jinzhong, China; ^2^ Key Laboratory of Land Surface Pattern and Simulation, Institute of Geographic Sciences and Natural Resources Research, Chinese Academy of Sciences, Beijing, China; ^3^ Department of Plant Breeding and Genetics, Sindh Agriculture University, Hyderabad, Pakistan; ^4^ College of Agriculture, Shanxi Agricultural University, Jinzhong, China; ^5^ School of Civil Engineering, Guangzhou University, Guangzhou, China

**Keywords:** Chlorophyll content, F_3_ segregating populations, genetic diversity, leaf area, rice (*Oryza sativa* L.), salinity tolerance, salt stress

## Abstract

Rice is an important cereal crop rich in starch and carbohydrates grown around the globe. Despite its significance, rice exhibits substantial genetic variation, particularly under environmental stresses such as salinity. This study investigates the genetic diversity of F3 segregating populations of rice under normal and salt stress. Various segregating genotypes were evaluated, demonstrating statistically significant differences (p<0.01 and p<0.05, ANOVA) in morphological and physiological parameters. The genotypes Kharagnjia and L-12 performed well in normal soils, while Shua-92 and L-20 showed better performance in tiller plant-1 and panicle length. The cluster analysis grouped rice genotypes into four major clusters based on genetic similarity. Principal Component Analysis (PCA) identified tillers per plant, panicle length, grain yield per plant, and leaf area as key contributors to genetic variation. The highest variability was observed in PC-XII (100%) and PC-XI (98.3%). These findings provide valuable insights for breeding programs aimed at enhancing salt tolerance in rice.

## 1 Introduction

Rice *(Oryza sativa* L.*)* (2n = 24) belongs to the family *Poaceae* and holds one fifth of the total area of land covered by cereal crops. It serves as a staple food for over 50% of the global population, particularly in Asia, where it provides 30–80% of daily caloric intake and is considered a major source of staple food for more than 50% of the world’s population (Lafiandra et al., 2014; Nayar, 2014; Amagliani et al., 2016; Kesari and Jamal, 2017). It is considered a cash crop and is the second staple food source, providing 30–80% of the daily calories to the population. The global population is growing, and food consumption is on the rise (Fukagawa and Ziska, 2019). It is considered a vital part of the diet of three billion people or more all around the globe and is considered the main source of various nutrients and intake of calories in progressive and developmental countries (Ritchie, 2021). It is essential to boost rice production to meet the rising food demands of the globe, as there is an expectation that the world’s population will touch the figure of 9.6 billion by 2050. Similarly, rice plays a significant role in global and regional demands for food security (Cuaton and Delina, 2022).

However, rice cultivation faces significant challenges due to abiotic stresses, with soil salinity being one of the most damaging (Shagun, 2024). Salinity affects around 45 million hectares of irrigated land worldwide, drastically reducing rice yield and quality. Salinity stress inhibits rice development via two main mechanisms: osmotic stress and ion toxicity. Osmotic stress, caused by high soil salt concentrations, reduces water absorption, leading to dehydration and stunted growth (Zhang et al., 2022a). Ion poisoning is caused by the buildup of sodium (Na^+^) and chloride (Cl^-^) ions in plant tissues, compromising cellular balance and metabolism (Alharbi et al., 2022). These negative consequences need to be mitigated in saline-prone locations (Oelviani et al., 2024). Therefore, it is crucial to develop salt-tolerant rice cultivars through genetic selection and breeding programs.

The genetic diversity in rice populations offered a range of traits (Na^+^/K^+^ ratio, salt injury score, shoot/root biomass, ion homeostasis, and stress-responsive gene expression) that can enhance salinity tolerance, and segregating populations, particularly F_3_ lines, were especially valuable (Raghuvanshi et al., 2021). F_3_ segregating populations play a crucial role in breeding programs as they provide a diverse genetic base for selecting desirable traits, including salinity tolerance. These populations allow researchers to assess genetic variability, identify stable genotypes, and select lines with superior agronomic performance under stress conditions. In this study, F_3_ populations were chosen because they represent an intermediate generation where genetic recombination has stabilized to some extent, making them ideal for evaluating salinity tolerance traits and their heritability. The findings from this study will contribute to breeding efforts aimed at developing salt-tolerant rice varieties by identifying promising genotypes with enhanced stress resilience.

Measurement of genetic variation commonly takes place by genetic similarity or genetic distance, based on which similarities and differences of the plant at the genetic level were identified. As rice is considered a staple food that feeds over half of the world, it is necessary to conduct different studies based on various parameters to unravel its genetics (Govindaraj et al., 2015). Different genetic combinations were expressed in these populations because of recombination across many generations. F_3_ populations under salt stress conditions help researchers find and select individuals with greater tolerance, leading to the development of robust rice cultivars. The basis of genetic variation analysis is the presence of variations in DNA sequences, as the genotypic variation genotypes come from the DNA sequence variations. Plant breeders are particularly interested in analyzing the level of genetic variation among rice cultivars (Sonsungsan et al., 2024).

Recent advancements in genetic approaches have significantly enhanced our understanding of salt tolerance in rice. The integration of nano-based strategies, such as nano-zinc oxide, has been shown to modulate stress-responsive gene expression and activate antioxidant defense mechanisms, thereby mitigating heavy metal stress in rice plants​ (Adil et al., 2024). Similarly, the application of selenium (Se) and brassinosteroids (BRs) has demonstrated promising results in improving photosynthetic efficiency, protein expression patterns, and overall plant resilience under abiotic stresses (Saeed et al., 2023; Khan et al., 2024). These compounds enhance antioxidant enzyme activities, reduce oxidative damage, and regulate key metabolic pathways, thereby strengthening plant defense mechanisms. Additionally, recent proteomic analyses have revealed key protein factors responsible for improving plant stress tolerance and nutrient utilization, highlighting novel molecular targets for genetic improvement (Ahmad et al., 2020). Incorporating these emerging biotechnological interventions into traditional breeding programs could pave the way for developing salt-tolerant rice cultivars with improved yield stability under salt stress.

The genetic diversity within rice populations offers a reservoir of traits that contribute to salinity tolerance. Segregating populations, particularly F3 lines, provide a unique opportunity to identify individuals with enhanced tolerance. Genetic diversity assessments rely on measures of genetic similarity and distance, revealing potential candidates for breeding. Given rice’s global importance, understanding its genetic variability under salt stress is essential for ensuring food security and sustainable agricultural practices. This study aims to assess genetic diversity in segregating populations under salt stress to identify superior genotypes for breeding.

## 2 Materials and methods

### 2.1 Experimental site and design

This study was conducted during the Kharif season of 2023 at the Students’ Experimental Farm, Department of Agronomy, Sindh Agriculture University, Tandojam. The climate in the research region was dry to semi-arid, with lengthy summers and moderate winters. The average temperature in the summer (April to September) was 31.8°C, and in the winter (October to March), it was 24.4°C, with an average relative humidity of 40%. The soil clay loam texture was detected in previous research in the same experimental field (Ali et al., 2024). The experiment followed a randomized complete block design (RCBD) with factorial arrangements and three biological replications. The field size was 15 × 15 square feet, with plant and row spacing of 20 cm each. Two salt treatments were applied (Chele et al., 2021).

- **Saline soil:** EC = 6.75 dS m^-1^, TSS = 1222.4 mg L^-1^.
- **Non-saline soil:** EC = 0.95 dS m^-1^, TSS = 608 mg L^-1^.

### 2.2 Plant materials

Salt tolerant genotypes were screened out from 20 genotypes and six genotypes with high yield, salt tolerant, and early plantation were chosen on a morpho-physiological basis and crossed under half diallel mating design, and F_1_ seed was raised. The first generation (F_1_) from these crosses was bulked and self to obtain F_2_ families, from which selected 10 lines were used under normal and saline field conditions (L-2, L-4, L-6, L-8, L-10, L-12, L-14, L-16, L-18, and L-20). The F_2_ families were advanced to produce F_3_ families. A random sample of F_3_ families derived from F_2_ individual plants was selected for evaluation. In addition to this, two parents, Shua-92 and Kharaganjia used as a check variety (Chen et al., 2024). Significant variations (p<0.01 and p<0.05) were observed among genotypes, treatments, and their interactions in F3 segregating rice populations, indicating the influence of morpho-physiological traits on yield (**Table 1**). Different characteristics affect yield under both saline and normal soils.

**Table 1:** Genotypes/F_3_ hybrids= 24 (saline=12, normal=12).

S. No	Genotypes grown in salt stress	Genotypes grown in non-saline/normal conditions
1	L-2	L-2
2	L-4	L-4
3	L6	L-6
4	L-8	L-8
5	L-10	L-10
6	L-12	L-12
7	L-14	L-14
8	L-16	L-16
9	L-18	L-18
10	L-20	L-20
11	Shua-92 (Check variety)	Shua-92 (Check variety)
12	Kharagnjia-92 (Check variety)	Kharagnjia-92(Check variety)

### 2.3 Morphological and physiological observations/measurements

The various morphological characters were studied in this experiment viz. plant height (cm), number of tillers plant^-1^, panicle length (cm), grains panicle^-1^, 1000-grainweight (g), grain yield plant^-1^ (g), biological yield plant^-1^ (g), harvest index (%). Some important physiological assays were also carried out using respective standard methods as described below:

#### 2.3.1 Leaf area (cm2)

The area of the leaf was determined using LI-COR equipment. A pressed leaf was put inside the equipment, and the leaf area was measured.

#### 2.3.2 Chlorophyll content (RG)

Leaves samples were collected and chlorophyll content was taken with the help of a SPAD meter. The top, mid, and bottom parts of the leaf were inserted inside the top portion of the SPAD, and the mean of each plant’s chlorophyll content was taken (Zhang et al., 2022b).

#### 2.3.3 Determination of total Na and K in rice leaves

The concentration of Na^+^ and K^+^ in rice leaves was determined through the 1:5 di-acid (HNO_3_- HCLO_4_) weight digestion method. Na^+^ and K^+^ readings were taken from the flame-photometer, and the final calculation was performed using the standard formula (Munns et al., 2010).

### 2.4 Statistical analysis

Statistical analysis was conducted using the Statistix v:8.1 software to analyse the data collected for each parameter. The analysis of variance (ANOVA) was determined by Gomez and Gomez (1984) to measure the overall significance of the data for variation. The means of the genotypes for all the characters were compared by using the least significant difference (LSD) at a 5% probability level. The cluster analysis was determined by using the squared Euclidian distance. PCA was performed using the multivariate statistical package (MVSP-3). Moreover, origin software was used to create graphical representations of the data, with the standard error (Freeman et al., 1985).

## 3 Results

### 3.1 Cluster analysis of various rice segregating populations

**Figure 1:**
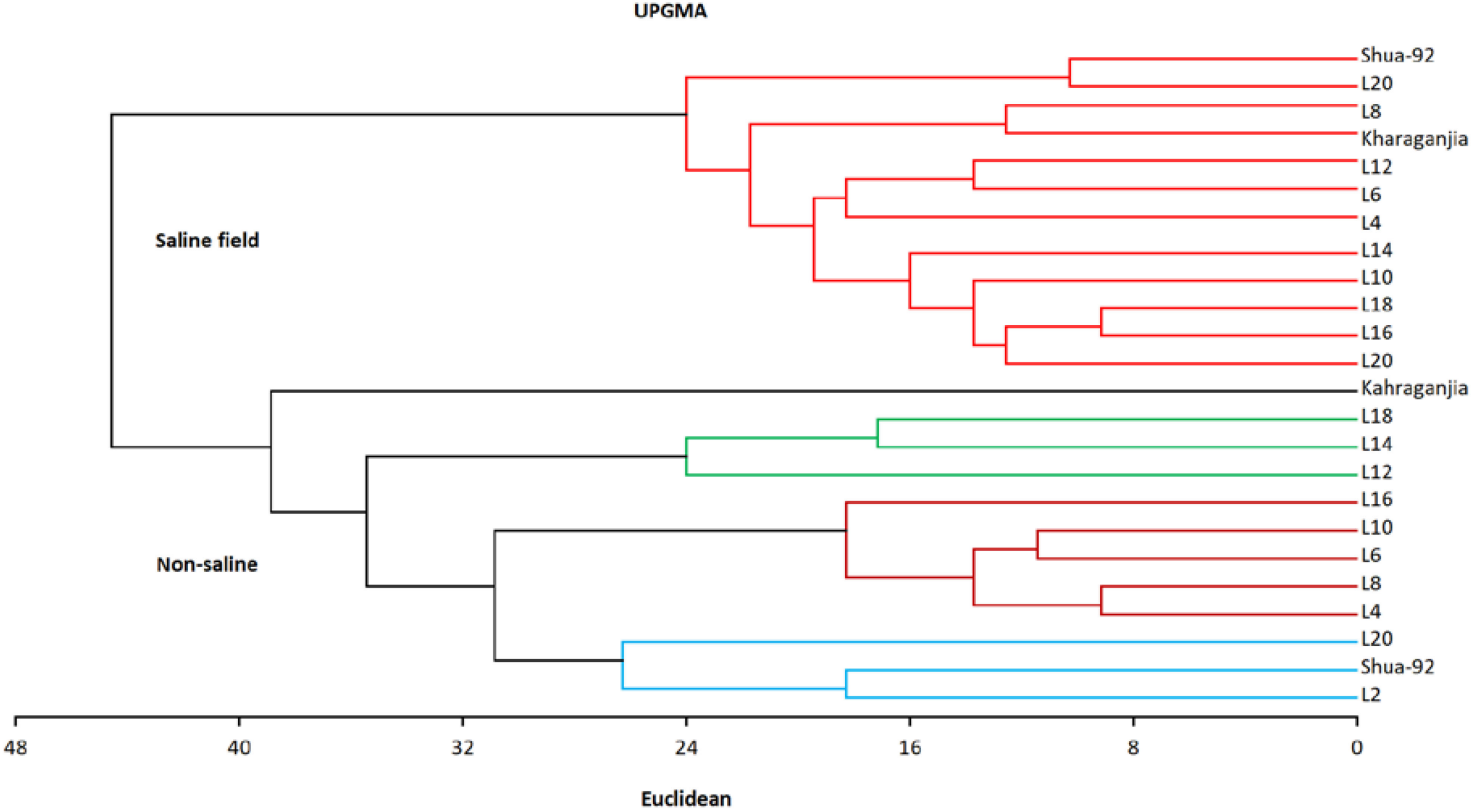
Dendrogram the relationship among different rice genotypes.

Cluster analysis groups genotypes into clusters based on genetic homogeneity, with high heterogeneity between clusters (Ahmed et al., 2017). A dendrogram based on Jaccard’s similarity values was constructed using the UPGMA cluster analysis. The separation of rice genotypes into four main groups according to their genotypic homogeneity takes place through cluster analysis. Cluster 1 contains twelve genotypes (Shua, L-20, L-8, Kharagnjia, L-12, L-6, L-4, L-14, L-10, L-18, L-16, L-12) and there was a close relationship among them. Cluster 2 included one genotype, namely Kharagnjia, whereas Cluster 3 contains genotypes L-8, L-14, and L-12, and Cluster 4 contains eight genotypes L-16, L-10, L-6, L-8, L-4, L-20, Shua and L-2 as shown in **Figure 1**.

### 3.2 Effects on performance based on yield of rice under salt stress

The presence of the same genotypes under contrasting soil conditions allowed for a direct comparison of their performance in response to salinity stress. The selected genotypes represented a diverse genetic pool, offering valuable insights into their tolerance and adaptability. The treatments significantly improved the physiological parameters, i.e., plant height, number of panicle plant^-1^, number of grains panicle^-1^, 1000 grains weight, and harvest index. Chlorophyll contents, potassium content (0.01003), and sodium content (0.07031), were also significantly elevated under the treatments applied (**Table 2**).

**Table 2:** Mean Squares for analysis of variance for different morphological and physiological parameters under non-saline and saline field in rice genotypes.

Morphological and physiological parameters/characters	Treatment df=1	Replication df=2	Genotypes df=12	Treatment x Genotype df=12	Error df=50
Plant height (cm)	15321.5**	102.7	131.0**	66.2*	30.2
Number of tillers plant^-1^	3.4892 ns	76.1303	38.0841 ns	30.2018 ns	37.5904
Number of panicle plant^-1^	2066.92**	0.19	116.77**	150.58**	2.60
Number of grains panicle^-1^	3002.22**	3.18	396.48**	308.03**	4.60
1000 grain weight (g)	595.930**	63.285	7.877*	5.703 ns	4.246
Biological yield plant^-1^(g)	0.189 ns	0.677	258.144**	401.931**	2.715
Grain yield plant^-1^(g)	11.7612*	2.1790	7.9505**	6.8929**	1.6525
Harvest index (%)	23.3472**	0.1918	35.1611**	8.8321**	1.7979
Chlorophyll content (RG)	53.9241**	8.9171	26.7823**	31.3171**	5.5784
Leaf area (cm^2^)	1.7329 ns	4.6262	11.5050 ns	18.0860 ns	18.0208
Potassium content	0.01003**	0.00124	0.00310**	0.00037**	0.00010
Sodium content	0.07031**	0.00015	0.01108**	0.00296**	0.00027

**significant at P<0.01probability level, *Significant at P<0.05 probability level and ns, Non-significant.

Significant differences were recorded across the genotypes, indicating the impact of salinity on key agronomic traits. Plant height, tiller number, panicle length, and grain yield per plant were notably reduced under salt stress, whereas genotypes such as L-2, L-20, and Kharagnjia exhibited comparatively better performance, suggesting their tolerance to salinity. Chlorophyll content and leaf area also declined under stress, highlighting the adverse effects of salinity on photosynthetic efficiency (**Table 3**).

**Table 3:** Mean performance of morphological parameters viz. plant height (cm), number of tiller plant^-1^ and panicle length (cm) under non-saline and saline field in rice genotypes.

Parents and hybrids	Plant height (cm)		R.D%	Number of tillers plant-1		R.D%	Panicle Length (cm)		R.D%
	Non-saline	Saline		Non- saline	Saline		Non-saline	Saline	
L-2	92.31	64.8	29.80	14.93	11.22	24.83	32.48	28.03	13.68
L-4	85.63	63.1	26.31	13.40	11.78	12.11	37.88	28.65	24.36
L-6	81.64	58.89	27.87	15.60	13.89	10.98	36.51	25.04	31.42
L-8	82.31	66.53	19.17	13.60	12.40	8.82	35.70	24.29	31.97
L-10	87.11	55.41	36.39	15.13	11.33	25.10	33.53	26.23	21.77
L-12	100.13	68.05	32.04	12.86	11.45	10.99	38.20	27.46	28.12
L-14	96.91	60.57	37.50	13.44	12.60	6.25	39.88	27.85	30.15
L-16	91.47	65.83	28.03	14.46	9.33	35.46	29.70	27.76	6.54
L-18	92.9	54.28	41.57	14.46	12.44	13.95	29.47	28.29	3.98
L-20	96.39	62.49	35.17	21.11	18.80	10.94	43.07	25.88	39.91
Shua-92	94.28	65.4	30.63	24.22	12.20	49.63	44.30	25.67	60.08
Kharagnjia	102.29	67.92	33.60	13.53	11.22	17.05	32.14	29.11	9.45
Average	91.95	62.77	31.51	15.56	12.39	18.84	37.74	27.02	25.12
LSD 5%	T= 2.6071	G= 6.3860	Tx G=9.0312	T=2.9089	G=7.1252	Tx G=10.077	T=0.7648	G= 1.8734	Tx G= 2.6494

**Figure 2:**
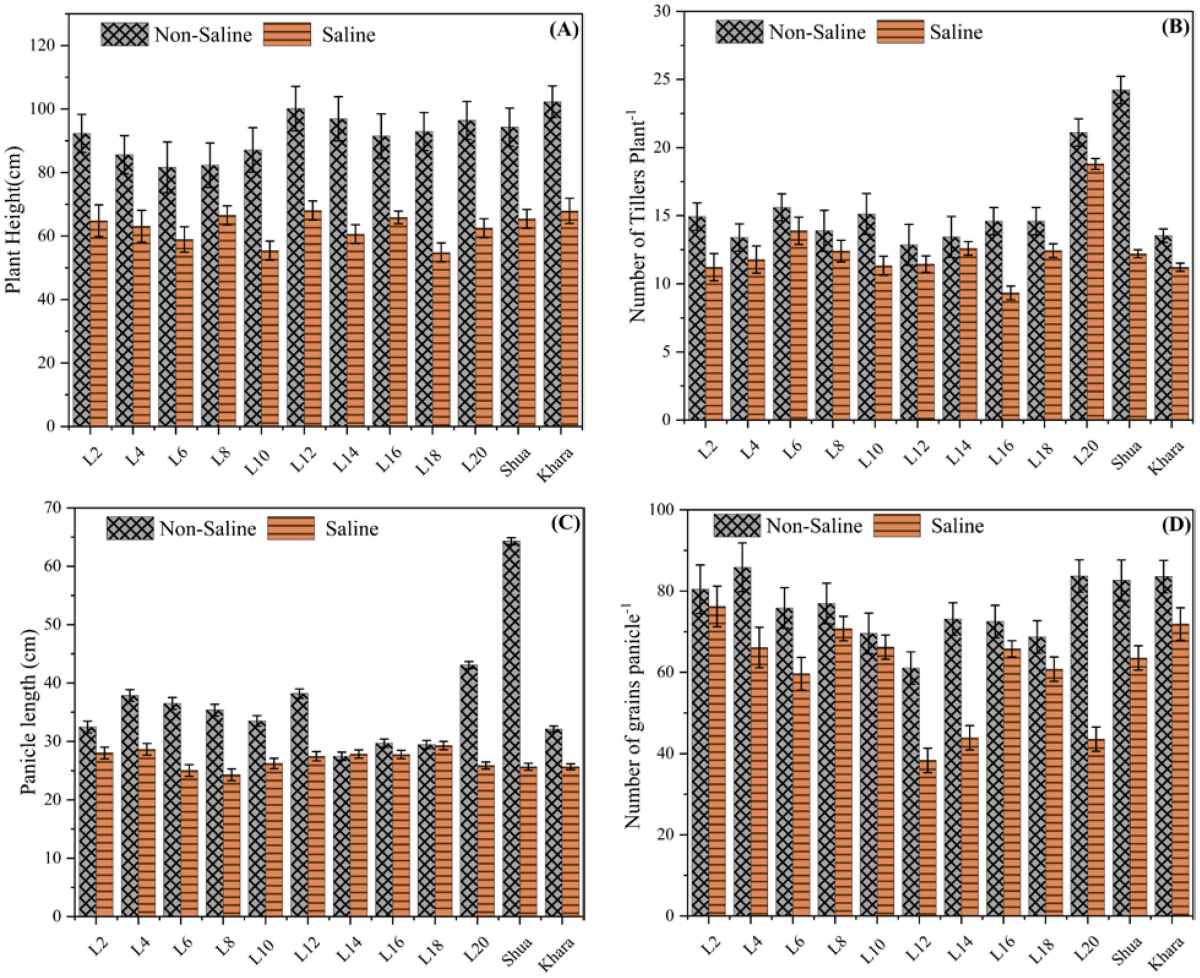
Comparison of morphological traits among different rice genotypes under saline and non-saline condition. The black-patterned bars represent the non-saline condition, while the solid bars indicate the salt stress. **(A)** Plant height (cm) across genotypes, with error bars representing variability and consistency across replicates. **(B)** Number of tillers per plant, ranging from 0 to 30, with error bars illustrating variability within the data. **(C)** Panicle length (cm) among different rice genotypes. **(D)** Number of grains per panicle under different environmental conditions.

**Figure 2** compares the plant height (measured in centimeters) under two different environmental conditions: non-saline (marked by a patterned bar) and saline (marked by a solid bar). The findings show a continuous pattern in which plant height was considerably higher in non-saline circumstances than in saline environments across all genotypes. For example, genotypes L12 and L14 depicted the largest plant height under non-saline circumstances, height approaching 100 cm or more. However, under saline circumstances, these genotypes showed a significant decrease in height (50-60 cm) (**Figure 2A**). Similarly, the genotypes L2 and L20 exhibited decreased growth in saline soils as compared to normal soil. The variations in plant height between genotypes under non-saline and saline soils indicate that salt has a deleterious influence on growth, with genotypic diversity in tolerance or susceptibility to salinity stress. Hence, from these findings, salt-tolerant genotypes may be efficiently provided.

The data reveals a consistent tendency across all genotypes: the number of tillers plant^-1^ was higher under non-saline soils than under saline environments. For example, genotype L20 produced maximum tillers under non-saline soils (25 tillers) ^-1^. As compared to check varieties Shua-92 and Kharaganjia showing considerable variations across non-saline and saline environments, In saline soils, the genotypes L12 and L14 showed a significant decrease in tiller production. In normal environments, these genotypes retained moderate tiller production (about 15–17 tillers) (**Figure 2B**). Most of the genotypes exhibited this pattern, which shows that salt has an adverse impact on tiller production. Genetic variations in salinity tolerance were evident from the differing degrees of reduction under saline conditions. Genotypes such as Shua-92 and L20, which maintain relatively higher tiller counts under saline stress, may be more resilient to salinity, whereas others exhibit a significant decrease in tiller production. Panicle length was affected under both saline and non-saline conditions for various genotypes (L2, L4, L6, etc.). The longest panicle length among the genotypes was found in Shua-92 under normal soil conditions, surpassing 60 cm. However, its panicle length experiences a significant reduction in salt stress, suggesting a high degree of sensitivity to salinity. Similarly, Kharaganjia exhibited a substantial decrease in panicle length when subjected to salt stress, although the difference was less pronounced than that observed in Shua-92 (**Figure 2C**). Minimum grain panicle^-1^ was found in genotypes under salt stress L-12 (38.33). However, L-2 (85.87) and L-20 (83.71) showed maximum grains panicle^-1^ under normal/non-saline field conditions, and minimum quantity was observed in L-12 (61.04). Grains panicle^-1^ was found greater in the genotypes SAL-2 along with Kharagnjia (check variety) at 76.22 and 71.88 under salt stress in (**Figure 2D**). In contrast, genotypes L2 to L20 had smaller panicle length differences between non-saline and salt stress, indicating greater resistance to salt stress than Shua-92 and Kharaganjia. These genotypes have more consistent panicle lengths, showing a decline under salt stress. Some genotypes, such as Shua-92 and Kharaganjia, were highly vulnerable to salinity stress, whilst others were quite resilient.

### 3.3 Manipulation in grain weight, biological, grain yield, and harvest index under saline and non-saline condition

Significant reductions in grain yield, 1000-grain weight, and biological yield were observed under salt stress. For instance, the grain yield per plant decreased from 25.6 g in non-saline conditions to 14.3 g under salt stress, highlighting the negative impact of salinity. The 1000-grain weight was also reduced, with genotypes like L-4 and L-6 maintaining relatively higher weights of 23.5 g and 22.8 g, respectively, compared to the lowest value of 17.2 g under saline conditions. Biological yield exhibited a similar trend, with a decline from 85.7 g in non-saline conditions to 56.4 g under salt stress. Despite these reductions, certain genotypes, such as L-4, L-6, and L-18, maintained relatively higher yields in both environments, suggesting their potential resilience. The harvest index also dropped from 35.2% under normal conditions to 28.1% in saline conditions, indicating a decline in resource-use efficiency (**Table 4**).

**Table 4:** Mean performance of morphological parameters viz. number of grains panicle^-1^, 1000 grain weight (g) and biological yield plant^-1^ (g) under non-saline and saline field in rice genotypes.

Parents and Hybrids	Number of grains panicle^-1^		R.D%	1000 grain weight (g)		R.D%	Biological yield plant^-1^		R.D%
	Non- saline	Saline		Non- saline	Saline		Non- saline	Saline	
L-2	80.45	76.22	5.26	22.75	12.53	44.89	78.33	62.19	20.60
L-4	85.87	66.11	23.01	19.00	14.64	22.97	96.4	77.84	19.24
L-6	75.86	59.66	21.36	18.19	11.64	36.01	93.91	87.29	7.049
L-8	76.97	70.77	8.06	20.00	14.13	29.33	96.49	79.19	17.92
L-10	69.57	66.21	4.83	17.46	13.22	24.29	91.88	71.44	22.24
L-12	61.04	38.33	37.21	21.24	13.88	34.67	91.6	84.43	7.82
L-14	73.16	43.89	40.01	20.20	14.77	26.89	79.25	67.02	15.43
L-16	72.50	65.78	9.27	18.96	13.28	29.96	83.50	80.96	3.04
L-18	68.72	60.78	11.56	18.36	12.63	31.21	76.50	71.06	7.11
L-20	83.71	43.55	47.98	16.40	13.15	19.81	92.88	66.08	28.85
Shua-92	82.66	63.55	23.12	19.69	16.45	16.46	87.22	67.91	22.14
Kharagnjia	83.55	71.88	13.97	20.47	13.38	34.64	87.41	71.49	18.21
Average	76.17	60.56	20.47	19.39	13.64	39.88	87.95	73.91	15.80
LSD 5%	T= 1.0181	G= 2.4937	Tx G=3.5267	T=0.9777	G=2.3948	Tx G=3.3867	T=1.9149	G=2.7080	Tx G= 0.6099

**Figure 3:**
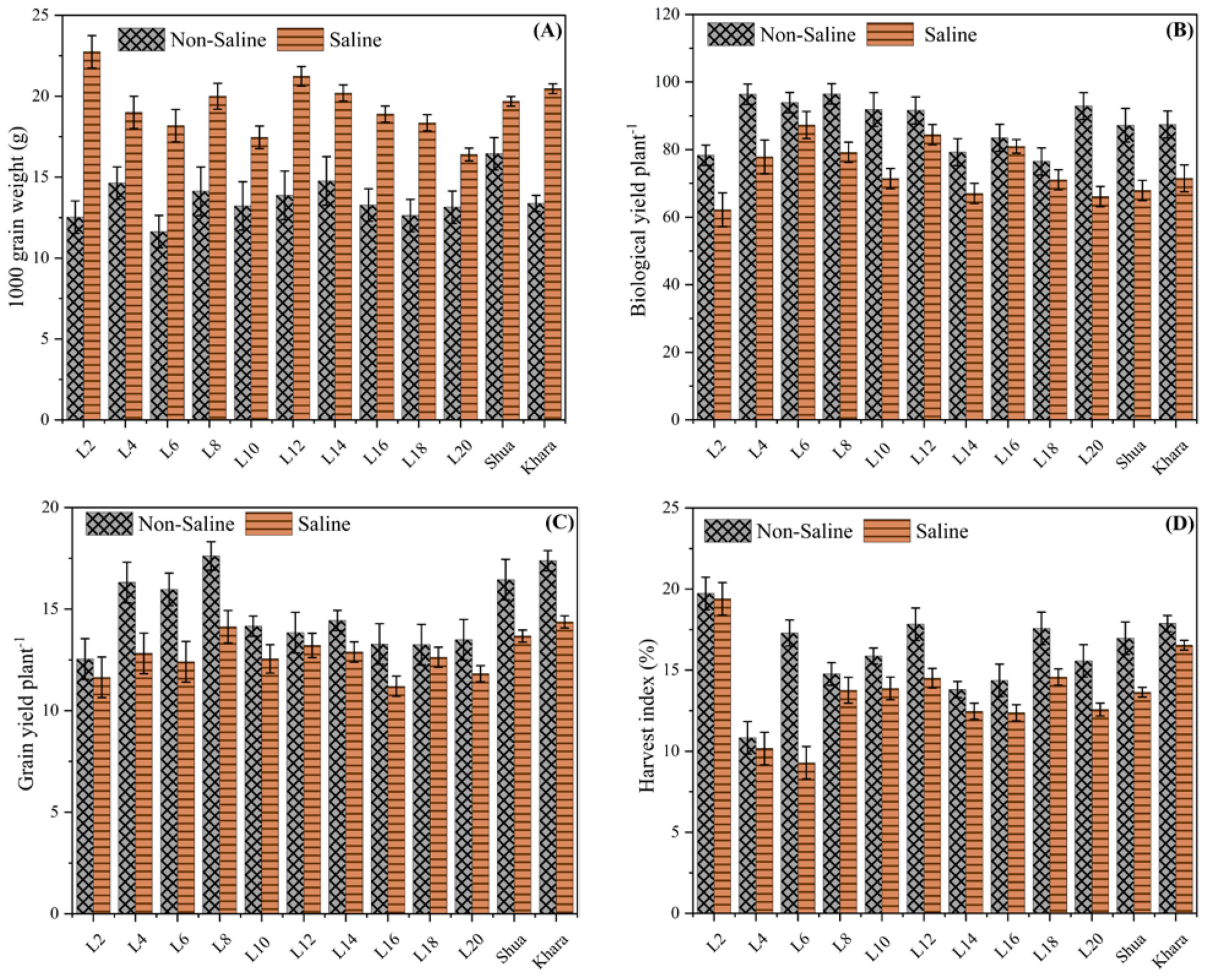
Comparison of yield-related traits among different rice genotypes under saline and non-saline condition. The black-patterned bars represent the non-saline condition, while the solid orange bars indicate the salt stress. **(A)** 1000-grain weight (g), showing higher values in salt stress, suggesting enhanced grain filling or size. **(B)** Biological yield per plant (g) across genotypes. **(C)** Grain yield per plant (g) under varying environmental conditions. **(D)** Harvest index (%), indicating the efficiency of biomass conversion into economic yield. Error bars represent variability within replicates.

**Figure 3** depicts the 1000-grain weights of several genotypes under saline and non-saline environments. Among the genotypes, L4, L12, and L18 had the maximum grain weight in saline soils, indicating their resilience or tolerance to salt stress. Conversely, the non-saline condition consistently results in lower grain weights across all genotypes, demonstrating a discrepancy in environmental effect. The cultivars Shua-92” and “Kharaganjia possessed comparatively greater grain weights in both environments, indicating their robustness and overall superior performance. This data implies that certain genotypes may be better adapted to salt stress, and more research might depict the biological yield plant^-1^ for different genotypes under both non-saline and saline environments (**Figure 3A**). This suggests that salt stress reduces total plant biomass, likely due to stress-induced growth restrictions. L4, L6, and L18 were the genotypes with the best yields under non-saline circumstances, indicating their potential for high production in ideal situations. In saline circumstances, yields were significantly reduced for all genotypes, with L4 and L18 doing substantially better than others, indicating a degree of tolerance or adaptation. Many of the genotypes (e.g., L4 and L18) performed well compared to Shua-92 and Kharaganjia, comparably under saline stress, indicating their robustness. The error bars represent variability, with specific genotypes having wider ranges, indicating higher irregularity in yield under stress. Overall, the findings showed that salinity has a considerable detrimental influence on biological output, emphasizing the necessity of developing genotypes that retain better productivity when stressed. In contrast to the trend in 1000-grain weight, biological yield was consistently greater in non-saline environments for all genotypes (**Figure 3B**). The grain yield plant^-1^ observed variations for various genotypes in both non-saline and saline situations. Genotypes L4, L6, and L18 performed well under normal soils, indicating a high yield potential. Yield was severely decreased under saline environments; however, certain genotypes, like L2, L4, and L6, depicted more tolerance, sustaining comparably larger grain yields as compared to Shua-92 and Kharaganjia (**Figure 3C**). The harvest index (%) of several genotypes (L2, L4, L6) under non-saline soils consistently show greater harvest indices than in salt stress, demonstrating how salinity has a negative impact on plant production. Under normal soils, L2 possessed the greatest harvest index (~20%), whereas, under salt stress, L6 exhibited the lowest (~5%). Salinity tolerance varied across the genotypes, with L2 and Shua-92 exhibiting the strongest tolerance (**Figure 3D**). These findings highlight the necessity for saline-tolerant cultivars to sustain output in difficult conditions.

### 3.4 Alterations in Chlorophyll content, leaf area, Na and K content under salt stress

A significant reduction in chlorophyll content was observed under salt stress, with values decreasing from 42.5 SPAD units in non-saline conditions to 30.8 SPAD units in saline conditions, indicating impaired photosynthetic efficiency. Leaf area followed a similar trend, declining from 45.3 cm² to 32.7 cm² under salt stress, suggesting restricted leaf expansion due to osmotic stress. Sodium (Na^+^) accumulation increased markedly in saline conditions, with the highest value recorded at 4.8 mg g^-^¹ DW, compared to 2.1 mg g^-^¹ DW in non-saline conditions, highlighting the detrimental ion imbalance caused by salinity. Conversely, potassium (K^+^) concentration declined from 3.9 mg g^-^¹ DW in normal conditions to 2.4 mg g^-^¹ DW under salt stress, indicating a disruption in ion homeostasis. Among the genotypes, L-2 and Kharagnjia exhibited higher chlorophyll retention and a better K^+^/Na^+^ ratio, suggesting their potential salt tolerance (**Table 5**).

**Table 5:** Mean performance of physiological parameters viz. sodium content (Na^+^) ratio and potassium content (K^+^) ratio under non-saline and saline field in rice genotypes.

Parents and Hybrids	Sodium content (Na+) ratio		R.D%	Potassium content (K+) ratio		R.D%
	Normal	Saline		Normal	Saline	
L-2	0.4700	0.4000	14.89	0.2500	0.2133	14.68
L-4	0.5600	0.5000	10.71	0.2767	0.2733	1.2
L-6	0.4667	0.4200	10	0.2267	0.2100	7.3
L-8	0.5233	0.5000	4.4	0.2500	0.2267	9.32
L-10	0.5300	0.4167	21.37	0.2700	0.2600	3.70
L-12	0.5600	0.5100	8.92	0.2500	0.2200	12
L-14	0.5400	0.5100	5.5	0.2700	0.2267	16.03
L-16	0.5400	0.5067	6.1	0.2767	0.2200	20.49
L-18	0.6000	0.4767	20.55	0.2700	0.2500	7.4
L-20	0.6133	0.4600	24.99	0.2900	0.2800	3.4
Shua-92	0.5400	0.5067	6.1	0.2700	0.2467	8.62
Kharagnjia	0.4333	0.4200	3.06	0.3000	0.2900	3.3
Average	0.53	0.47	11.38	0.27	0.24	8.95
LSD 5%	T= 7.862	G=0.0193	Tx G=0.0272	T= 4.773	G= 0.0117	Tx G=0.0165

**Figure 4:**
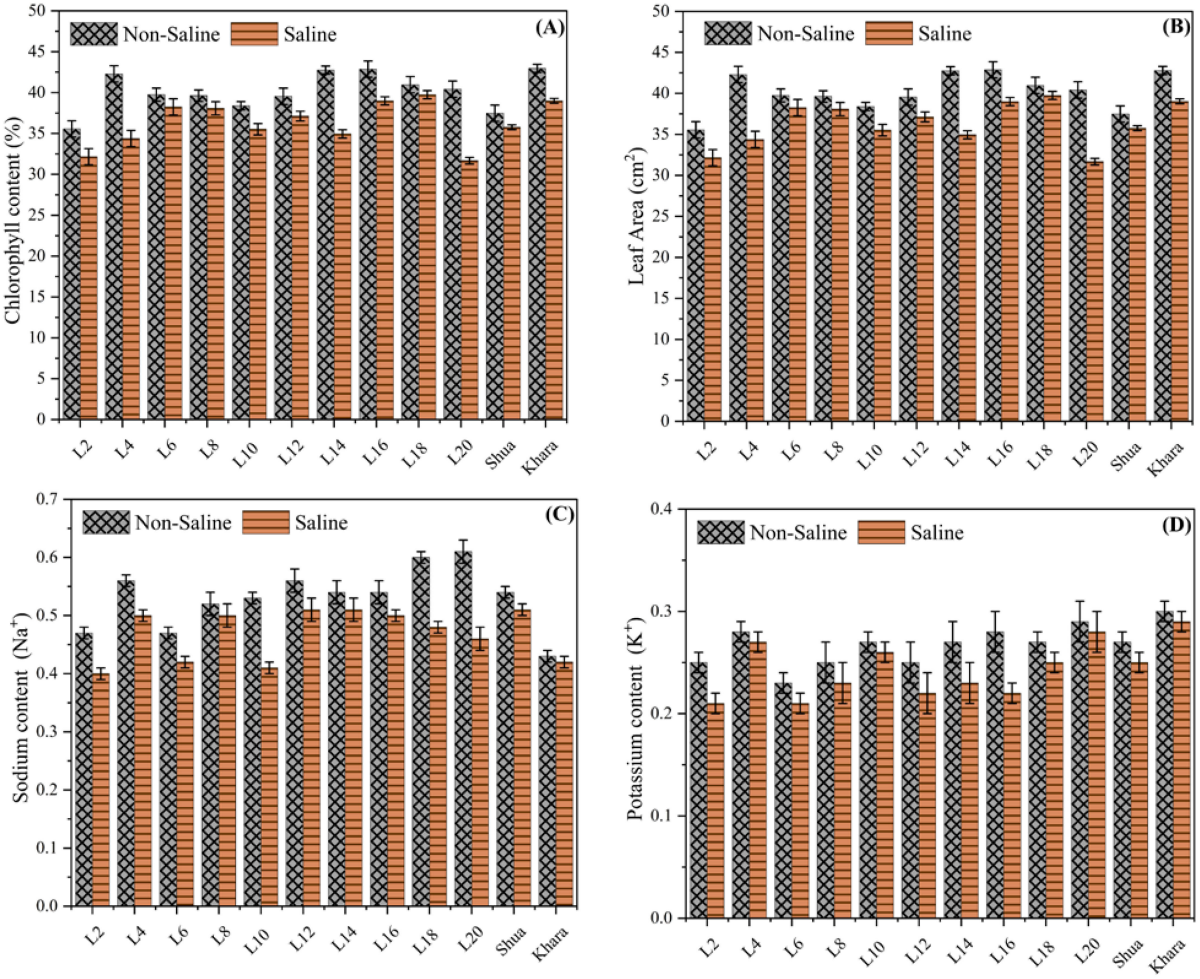
Chlorophyll content vs different genotypes of rice. The chlorophyll content (%) of different genotypes (L2, L4, L6, etc.) in non-saline (crosshatched bars) and saline (solid bars) conditions **(A)**. The leaf area (cm²) of various genotypes (L2, L4, L6, etc.) was compared under saline (solid bars) and non-saline (crosshatched bars) conditions **(B)**. The Sodium Ratio of variability among different genotypes **(C)**, and Potassium level of variability among different genotypes **(D)**.

**Figure 4** shows that under non-saline condition, L2 exhibited the highest chlorophyll content (approximately 45%), while under salt stress, it reduced to approximately 35%. Variability among genotypes was evident in both conditions, with L2 and Shua-92 exhibiting superior chlorophyll retention during saline-induced stress. The chlorophyll content of genotypes such as Kharaganjia was substantially reduced under salt stress, which emphasizes their susceptibility. These results indicate that genotypes with greater chlorophyll retention under salinity, such as L2, may be advantageous for breeding initiatives designed to enhance crop tolerance to salinity, as shown in (**Figure 4A**). The leaf area was consistently greater in non-saline condition than in saline soils, suggesting that salinity has a detrimental impact on plant growth. Under non-saline condition, L2 exhibits the highest leaf area (~45 cm²), which considerably decreases to approximately 40 cm² in salt stress. Genotypes such as Kharaganjia sustain a comparatively high leaf area under salinity, indicating a higher tolerance level. Conversely, genotypes like L6 exhibit significant reductions in leaf area in saline environments. The adverse impact of saline environments on the overall vigor of plants and the photosynthetic capacity was underscored by the reduction in leaf area under salinity. To maximize growth and productivity under salt stress, genotypes with higher leaf area retention, such as Kharaganjia and L2, could be targeted for breeding salinity-tolerant crops (**Figure 4B**). The concentration of sodium (Na^+^) among genotypes varied under both saline and non-saline condition. L2, L4, L6, and so forth, including Shua-92 and Kharaganjia, denoted distinct interventions, under non-saline condition, L12 and L18 exhibited maximum sodium levels, while L2, L4, and Shua-92 exhibited relatively decreased sodium content. In salt stress, the sodium levels were consistently reduced across all samples, with L12 and L18 still maintaining comparatively higher Na^+^ compared to others. Under both circumstances, Shua-92 and Kharanjia ra demonstrated moderate sodium levels. Wholly, whole, the findings showed that under saline soils dramatically lower salt absorption in all genotypes were found, pointing to a putative stress response or control mechanism. Variations in sodium levels across samples may indicate variations in salt tolerance or absorption efficiency (**Figure 4C**). ‘Under normal soils L2, L4, and L6, including Shua-92 and Kharaganjia, reflected changes in potassium levels under both circumstances. Revealed that under non-saline soils, L6, L12, and L18 substantially possessed greater potassium concentrations, whereas L2 and L20 were found to have moderate amounts. In saline soils, potassium levels decreased significantly in most of the genotypes, with L4, Shua-92, and Kharaganjia ra retaining somewhat more potassium than others (**Figure 4D**). These findings showed that saline soils have a deleterious influence on potassium, which was necessary for cellular activities and stress tolerance. Differences across samples may indicate variable levels of tolerance or regulatory mechanisms influencing potassium absorption under salinity stress.

### 3.5 Principal component analysis

The first principal component (PC-1) accounted for the highest variance (26.7%) and was primarily influenced by traits such as tillers per plant, panicle length, grain yield per plant, and leaf area. The second principal component (PC-2) explained 18.4% of the variation, with strong contributions from plant height, grains per panicle, and 1000-grain weight. The cumulative variance reached 98.3% by PC-11, with PC-12 capturing the remaining 1.7%, demonstrating that the first few components effectively summarized the variability among genotypes. Notably, traits such as harvest index, chlorophyll content, and sodium and potassium accumulation played significant roles in later components, highlighting their influence on stress tolerance (**Table 6**).

**Table 6:** Principal components (PCs) analysis for various morpho-physiological traits in rice genotypes.

	PC 1	PC 2	PC 3	PC 4	PC 5	PC 6	PC 7	PC8	PC 9	PC 10	PC 11	PC 12
Eigen values	3.20	1.61	1.27	1.20	0.99	0.90	0.73	0.67	0.45	0.40	0.32	0.20
Percent of variance	26.7	13.4	10.6	10.0	8.3	7.5	6.1	5.7	3.8	3.4	2.7	1.7
Cumulative Percentage	26.7	40.1	50.8	60.8	69.1	76.6	82.7	88.4	92.2	95.6	98.3	100.0

**Figure 5:**
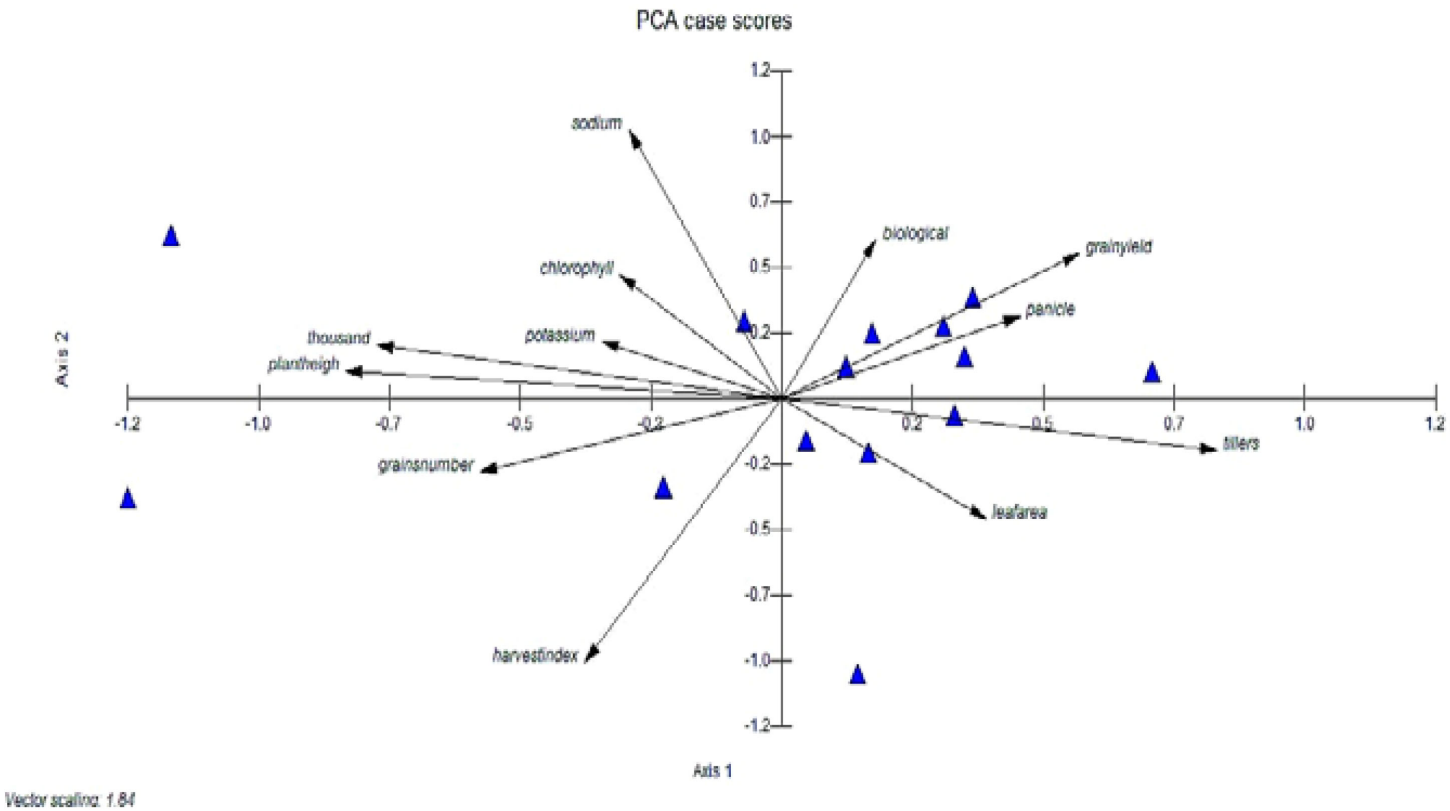
Genetic diversity using PCA score of different rice genotypes, PCA was used to analyze the mean data. The significance of the main contributor to the total variance at each differentiation axis.

The eigenvalues are often used to determine how many factors to retain (Kanavi et al., 2020). Different morpho-physiological characteristics were studied through PCA to determine their relationship within these parameters. The presence of both positive and negative correlation trends within the variables and components was shown by positive and negative loadings. Thus, tillers plant^-1^, panicle length, grain yield plant^-1^, and leaf area had high positive component loadings for the first principal component (PC-I), while plant height, 1000-grain weight, biological yield, harvest index, chlorophyll content, and sodium and potassium content had high negative loadings. In the second principal component (PC-II), high positive component loading was observed from plant height, grains panicle^-1^, 1000-grain weight, harvest index, and leaf area, whereas it had high negative value for tillers plant^-1^, grains panicle^-1^, biological yield plant^-1^, grain yield plant-1, chlorophyll content, sodium, and potassium content. Tillers plant^-1^, grains panicle-1, biological yield plant^-1^, harvest index, chlorophyll content, and leaf area were the main contributing characters to the variation in the third principal component (PC-III). Plant height, panicle length, 1000-grain weight, grain yield plant^-1^, sodium, and potassium content showed high negative values for these traits, while harvest index, chlorophyll content, and leaf area showed high values for positive loadings. Likewise, strong positive component loading from harvest index, chlorophyll content, and grains panicle^-1^ was noted in the fourth principal component (PC-IV), whereas it was negative from plant height, tillers plant^-1^, panicle length, 1000-grain weight, grain yield plant^-1^, biological yield plant^-1^, leaf area, sodium and potassium content. The fifth principal component possessed high PCA loading in plant height, 1000-grain weight, biological yield plant^-1^, grain yield plant^-1^, harvest index, chlorophyll content, and leaf area, whereas the highest negative was observed in tillers plant^-1^, panicle length, grains panicle^-1^, sodium, and potassium content. In the sixth (PC-VI) were plant height, tillers plant^-1^, 1000-grain weight, biological yield plant^-1^, and sodium content, while the negative loading factors were observed in panicle length, grains panicle^-1^, grain yield plant^-1^, harvest index, chlorophyll content, leaf area, and potassium content. The seventh principal component showed maximum loadings in plant height, tillers plant^-1,^1000-grain weight, panicle length, grain yield plant^-1^, harvest index, chlorophyll content, and sodium content, while the negative loading factors were observed in grains panicle^-1^, biological yield plant^-1^, leaf area and potassium content. The eighth principal component showed maximum loadings in panicle length, chlorophyll content, leaf area, sodium and potassium content, while the negative loading factors were observed in plant height. tillers plant^-1,^1000-grain weight, grain yield plant^-1^,harvest index, biological yield plant^-1^ and grains panicle^-1^. The ninth principal component showed maximum loadings in grains panicle^-1^, grain yield plant^-1^, leaf area and sodium content Whereas the negative loading factors were observed in plant height. tillers plant^-1,^1000-grain weight, harvest index, biological yield plant^-1^, chlorophyll content and potassium content. The tenth principal component showed maximum loadings in plant height and grain yield plant^-1^ whereas the negative loading factors were observed in tillers plant^-1^, panicle length, 1000-grain weight, harvest index, biological yield plant^-1^ and grains panicle^-1^, chlorophyll content and sodium. The eleventh principal component showed maximum loadings in plant height, panicle length, grains panicle^-1^, harvest index, biological yield plant^-1^, grain yield plant^-1^, sodium and potassium content whereas the negative loading factors were observed in tillers plant^-1^, 1000-grain weight, chlorophyll content and leaf area. The twelfth principal component showed maximum loadings in tillers plant^-1^, 1000-grain weight, harvest index, biological yield plant^-1,^grains panicle^-1^, grain yield plant^-1^, leaf area, sodium and potassium content whereas the negative loading factors were observed in plant height, panicle length and chlorophyll content. The first principle component showed the highest eigenvalue having 3.20. While others as PC-II, PC-III, PC-IV, PC-V, PC-VI, PC-VII, PC-VIII, PC-IX, PC-X, PC-VI and PC-XII showed lesser Eigen value symmetrically as shown in (**Figure 5**). The highest variability was accumulated in PC-XII and PC-XI which showed 100% and 98.3% variability and the least variability was observed in PC-I with 26.7% among different traits of rice accessions.

The clustering pattern observed in the dendrogram reflected the genetic and physiological responses of the selected rice genotypes to salinity stress. The genotypes grouped together exhibited similar tolerance or sensitivity traits, likely influenced by shared genetic backgrounds, physiological adaptations, and stress-responsive mechanisms. For instance, the salt-tolerant genotypes clustered together due to their enhanced antioxidant enzyme activities, efficient ion homeostasis, and lower Na^+^ accumulation in shoot tissues. In contrast, the salt-sensitive genotypes formed a separate cluster, characterized by higher Na^+^ uptake, reduced chlorophyll content, and impaired growth under salt stress. These findings demonstrated that salinity tolerance in rice is governed by a combination of genetic and physiological factors, contributing to distinct clustering patterns among genotypes.

## 4 Discussions

The genotypic responses to salinity stress varied significantly in terms of key morphological and physiological traits. Under saline conditions, salt-tolerant genotypes, such as L4 and L18, maintained higher plant height, greater tiller number, and longer panicles compared to sensitive genotypes. These genotypes also exhibited superior physiological adaptations, including a higher K^+^/Na^+^ ratio, better chlorophyll retention, and enhanced antioxidant enzyme activities, which contributed to their resilience. In contrast, salt-sensitive genotypes showed significant reductions in plant height, tiller number, and grain yield, along with higher Na^+^ accumulation, increased oxidative stress, and lower photosynthetic efficiency. These findings highlight the critical traits that distinguish salt-tolerant from salt-sensitive genotypes and provide valuable targets for future breeding efforts.

Principal component analysis (PCA) reduces multiple correlated variables to a smaller number known as principal components. Genetic diversity influences complicated attributes such as grain yield, which numerous genes regulate (Ahmad et al., 2014; Akram et al., 2014; Bashir et al., 2016). UPGMA revealed that these genotypes formed four distinct clusters. The genotypes formed different clusters. A dendrogram based on Jaccard’s similarity values was constructed using the UPGMA cluster analysis. Cluster analysis separated the rice genotypes into four main groups according to their genotypic homogeneity. Cluster 1 contains twelve genotypes (Shua-92, L-20, L-8, Kharagnjia, L-12, L-6, L-4, L-14, L-10, L-18, L-16, L-12) and there was a close relationship among them. Cluster 2 included one genotype, namely Kharagnjia; cluster 3 contains genotypes L-8, L-14 and L-12 and Cluster 4 contains eight genotypes L 16, L-10, L-6, L-8, L-4, L-20, Shua-92 and L-2 (**Figure 1**). Scientists led an examination to ponder the phenotypic variety and hereditary decent variety, significant elements in choosing germplasm for rice rearing (Roy et al., 2024). The phenotypic relationship demonstrated that many of the characters assessed the significance of the choice of high-yielding genotypes. These important features can be used effectively in selection to enhance rice accessions. For any breeding effort to be successful, a thorough understanding of the genetic diversity of the traits under consideration was necessary. Understanding the genetic factors of different yield and yield-contributing traits was essential to facilitate designing an efficient breeding strategy.

Different quantitative and qualitative characteristics were studied through PCA to get the outline relationship among these parameters. The loading plot (**Figure 5**) depicted the liaison of these parameters with each other. These results were consistent with those of (Zhen et al., 2018; Hussain et al., 2023; Yousaf et al., 2024), who reported that the tillers plant^-1^, panicle length, grain yield plant^-1^, and leaf area had high positive component loadings for the first principal component (PC-I), while plant height, 1000-grain weight, biological yield, harvest index, chlorophyll content, and sodium and potassium content had high negative loadings. In the second principal component (PC-II), high positive component loading was observed from plant height, grains panicle^-1^, 1000-grain weight, harvest index and leaf area whereas it had high negative value for tillers plant^-1^, grains panicle^-1^, biological yield plant^-1^, grain yield plant^-1^, chlorophyll content, sodium and potassium content. Therefore, to identify efficient genotypes that possess an appropriate trait combination, a variety of selection criteria must be implemented. Furthermore, the identification of the genetic variety within a genotype group was crucial in the development of superior recombinants. Conversely, principal component analysis (PCA) effectively identifies a set of reproductive lines that promotes the highest degree of diversity (Ahmad et al., 2021b; Akbar et al., 2023).

Cluster analysis grouped genotypes into four clusters based on genetic homogeneity. Cluster 1 contained 12 genotypes exhibiting close relationships, while Clusters 2, 3, and 4 exhibited distinct genetic variations. These findings align with previous studies on genetic diversity in rice under stress conditions. PCA identified tillers per plant, panicle length, grain yield per plant, and leaf area as the primary contributors to genetic variation. The first principal component (PC-I) accounted for the highest variance (26.7%), with cumulative variance reaching 100% in PC-XII. These findings indicate that the evaluated traits significantly influence salinity tolerance and should be prioritized in breeding programs.

Salinity tolerance varied across genotypes, which can be attributed to differences in key physiological and biochemical mechanisms. The salt-tolerant genotypes exhibited enhanced ion homeostasis, as indicated by lower Na^+^ accumulation and higher K^+^/Na^+^ ratios in shoot tissues, which is a well-documented trait in salt-tolerant rice varieties (Yasin et al., 2018; Akram et al., 2019). Additionally, these genotypes maintained higher chlorophyll content and photosynthetic efficiency under salt stress, suggesting better osmotic adjustment and oxidative stress management. Increased antioxidant enzyme activities, including superoxide dismutase (SOD) and catalase (CAT), were also observed in tolerant genotypes, which likely contributed to reduced oxidative damage. Conversely, salt-sensitive genotypes accumulated excessive Na^+^ in leaf tissues, leading to membrane damage, lower chlorophyll retention, and a significant reduction in plant biomass and grain yield under salinity stress. These results align with previous studies demonstrating that salinity tolerance in rice is primarily governed by mechanisms such as ion regulation, osmoprotection, and antioxidant defense responses.

Rice is significant in providing food to more than half of the world’s population (Alam et al., 2024). Salt stress affects 20% of global cultivable land and continuously increases due to climate change and anthropogenic activities (Hasanuzzaman and Fujita, 2022). Environmental stress, including salinity, can cause about 50% of production losses (Ahmad et al., 2022a; Sadak et al., 2022). The research aimed to provide hybrids with salt tolerance as it is now the basic need of our lands and production due to climatic and soil issues. Significant results were declared (*p*<0.01) under both normal and saline soils with various morpho-physiological characteristics. Similarly, enhanced yield was observed and positively correlated with various traits (Hoque et al., 2023). The superior lines evaluation and selection play an important role in producing ideal hybrid combinations (Khan et al., 2023). Mean performance results for plant height and for the number of tillers plant^-1^ suggested that the genetic materials possessed distinctive variations that could be used to develop new rice genotypes for further breeding programs (Rahman et al., 2016; Khanna et al., 2024). Our results were similar to Shrestha et al. (2021) and Lakshmi et al. (2021), who also reported significant variations among the genotypes for various traits in rice genotypes under saline and normal field conditions (Lakshmi et al., 2021; Shrestha et al., 2021). Rice genotypes showed larger panicle length, having more grains in the genotype in L-2 than in Kharaganjia under saline soils. Such results displayed that variable responses of rice genotypes to salinity stress depend on their growth stages (Hassan et al., 2023; Singh et al., 2023; Thakur et al., 2023). Under salinity stress, plant growth, tiller number, biomass accumulation, panicle number, spikelets panicle^-1^, grain-filling percentage, and grain yield all significantly declined (Ahmad and Liu, 2022; Alam et al., 2024). This decrease was attributed to ion toxicity, nutrient deficiency, and oxidative stress caused by salinity conditions (Sadak et al., 2022). The physiological characteristics displayed fluctuations under salt stress and normal soils and a decrease in chlorophyll content becomes a first indication of responses in plants subjected to salinity stress (Cheabu et al., 2024). When rice encounters salt stress, the growth was significantly limited and results in a low leaf area index (LAI) (Akram et al., 2022; Hussain et al., 2024). Na^+^ content was found higher in genotypes L-20 and L-18 describing that they were salt sensitive (Chen et al., 2021). The K^+^ content was found more in some tolerant rice genotypes and less affected as L-20 along with check Kharagnajia. Less K^+^ content was reflected, with some genotypes showing sensitivity in the crop (**Figure 4D**). Ionic toxicity was caused by salt stress, resulting in a large accumulation of intracellular Na^+^, disrupting the original ionic balance, causing a nutrient deficiency, and stunting plant growth and development (Ahmad et al., 2022b). More sodium accumulates in salt-sensitive rice, which impairs a wide range of cellular metabolisms, including photosynthesis, protein synthesis and lipid metabolism (Xie et al., 2020; Ahmad et al., 2021a).

Salinity reduced plant height, tiller number, and panicle length across all genotypes. However, L-2, L-20, and Kharagnjia retained higher grain yields under stress, demonstrating superior tolerance. Physiological responses such as chlorophyll retention and balanced Na^+^/K^+^ ratios correlated with stress resilience, suggesting potential mechanisms for tolerance (Zhen et al., 2018; Hussain et al., 2023; Yousaf et al., 2024). These findings provide valuable insights for breeding high-yielding, salt-tolerant rice varieties. Breeders’ interest in creating genetic variety has increased significantly in recent years (Ahmad et al., 2021c). Crossbreeding remained one approach for obtaining genetic diversity or introducing desired features into a new population. Lines that exhibit phenotypic variation, yield potential, and salinity stress tolerance. Identifying suitable lines might help generate early, high-yielding, salt-tolerant rice cultivars (Ahmad et al., 2021b; Akbar et al., 2023). The findings of this study have significant implications for rice breeding programs aimed at improving salinity tolerance. The superior-performing genotypes, such as Shua-92, L4 and L18, demonstrated key traits associated with salt stress resilience, including higher K^+^/Na^+^ ratios, greater chlorophyll retention, and enhanced antioxidant enzyme activities. These traits should be prioritized in breeding efforts to develop new salt-tolerant rice varieties. Additionally, genotypes exhibiting stable grain yield under salt stress conditions should be further evaluated for their genetic basis of tolerance. Future breeding programs can integrate these traits using marker-assisted selection (MAS) or genomic selection approaches to accelerate the development of high-yielding, salt-tolerant rice cultivars. Furthermore, the identification of salt-responsive physiological and biochemical markers in this study provides a valuable framework for screening rice germplasm, facilitating the selection of resilient genotypes for large-scale breeding trials.

While this study provides valuable insights into the genetic and physiological mechanisms of salinity tolerance in rice, certain limitations should be acknowledged. First, the study was conducted under controlled conditions, which may not fully replicate the complexity of field environments where multiple stress factors interact. Future research should validate these findings in diverse field conditions to assess genotype performance under real-world agricultural settings. Second, while we identified key physiological and biochemical traits associated with salinity tolerance, further molecular investigations, such as transcriptomic and proteomic analyses, could provide deeper insights into gene expression patterns and regulatory networks underlying salt stress responses. Additionally, integrating marker-assisted selection (MAS) and genome-wide association studies (GWAS) could facilitate the identification of genetic loci governing salt tolerance, aiding in more efficient breeding strategies. Future research should also explore the long-term stability of salt-tolerant traits across different growth stages and environmental conditions to ensure their applicability in large-scale breeding programs.

## 5 Conclusion

This study highlights the significant genetic variation among F3 rice populations under salt stress. The results emphasize the importance of genotypic selection for breeding programs aimed at improving salinity tolerance. Genotypes L-2, L-20, and Kharagnjia emerged as promising candidates for cultivation in saline-prone regions. Future research should focus on molecular characterization of salt-tolerant traits and their integration into breeding programs.

## Data Availability

The raw data supporting the conclusions of this article will be made available by the authors without undue reservation.
